# Hands-On Practice on Sustainable Simulators in the Context of Training for Rural and Remote Practice Through a Fundamental Skills Workshop

**DOI:** 10.7759/cureus.28840

**Published:** 2022-09-06

**Authors:** Samyah Siraj, Mithusa Sivanathan, Sandy Abdo, Julia Micallef, Bruno Gino, Dania Buttu, Krystina M Clarke, Marvin Mnaymneh, Andrei Torres, Gordon Brock, Carla Pereira, Adam Dubrowski

**Affiliations:** 1 Health Sciences, Ontario Tech University, Oshawa, CAN; 2 Discipline of Emergency Medicine, Memorial University of Newfoundland, St. John's, CAN; 3 Education, Ontario Tech University, Oshawa, CAN; 4 Computer Science, Ontario Tech University, Oshawa, CAN; 5 Family Practice, Centre De Santé De Témiscaming, Temiscaming, CAN; 6 Allergy and Immunology, Uberlândia Medical Center, Uberlândia, BRA

**Keywords:** clinical skills, hands-on practice, healthcare professionals, physicians, rural and remote, additive manufacturing, 3d printing, simulation-based education, simulator, training

## Abstract

Simulation-based education (SBE) is a sustainable method to allow healthcare professionals to develop competencies in clinical skills that can be difficult to maintain in rural and remote settings. Simulation-based skills training is necessary for healthcare professionals that experience difficulties accessing skills development and maintenance courses to address the needs of rural communities. However, simulators, a key element of simulation, are often prohibitively expensive and follow a “one-size-fits-all” approach. Using additive manufacturing (AM) techniques, more specifically three-dimensional (3D) printing, to produce inexpensive yet functional and customizable simulators is an ideal solution for learners to practice and improve their procedural skills anywhere and anytime. AM allows for the customization of simulators to fit any context while reducing costs and is an economic solution that moves away from the use of animal products to a more ethical, sustainable method for training. This technical report describes the delivery of a fundamental skills workshop to provide hands-on training to rural and remote healthcare professionals using 3D-printed simulators purposefully designed following design-to-cost principles. The workshop was delivered at a three-hour session hosted at a rural and remote medicine course in Ottawa, Canada. The workshop consisted of four technical skills: suturing, cricothyrotomy, episiotomy, and intraosseous infusion (tibial) (IO) and used a blended learning approach to train healthcare professionals and trainees who practice in rural and remote areas. In addition, the learners were granted access to a custom-designed learning management system, which provided a repository of instructional materials, and enabled them to record and upload personal practice sessions, review other learners' practice sessions, collaborate, and provide feedback to other learners. The feedback collected from participants, instructors, and observations on the delivery of the workshop will help improve the structure and training provided to learners. The delivery of this workshop annually is an ideal solution to ensure parsimonious delivery of simulation training for rural and remote healthcare professionals.

## Introduction

The shortages of healthcare professionals practicing in rural communities and a low retention rate impact Canadians who present with poor health and medical emergencies in rural and remote settings [1]. In addition, the healthcare professionals that stay in rural and remote contexts experience difficulties accessing skills development and maintenance courses to address the diverse health needs of rural communities due to factors such as distance from urban centers and cost. Therefore it is crucial to provide them the appropriate simulation-based skills training [2]. Continuing medical education is one mechanism that allows for lifelong learning through conferences, courses, workshops, and more so that healthcare professionals can maintain the knowledge and skills necessary for practice [3].

Simulation-based education (SBE) is a sustainable method to allow healthcare professionals to practice and develop competence in clinical skills that are acquired through consistent practice. The use of SBE provides learners with the opportunity to practice skills in a safe environment, where making a mistake in practice will not put a patient's safety at risk [4-5]. Additive manufacturing (AM), which is a computer-controlled process that creates three-dimensional objects, is disrupting SBE [6]. Three-dimensional (3D) printing is the most known form of AM. The benefits of AM include the customization of simulators to fit any context and the reduction of costs [7]. By allowing practice on 3D-printed simulators, learners can improve their procedural skills [8]. 3D-printed simulators are not only reproducible but also allow the learner to get a near realistic and accurate feel when practicing a clinical skill [9]. 

In rural areas, learners practice clinical skills on cadavers, live animals, or animal parts such as pig feet in authorized hospitals and educational institutions, which are costly to maintain and not reusable [10]. AM techniques are an economic solution that moves away from the use of animal products to a more ethical, sustainable method for training. The design-to-cost approach to development focuses on reducing costs by sacrificing part of the realism of the model to make the simulator more accessible for learners. Conversely, the design-to-value approach aims to deliver a more anatomically correct model to the learner, which may increase the production cost but will deliver a more accurate representation of a real-life procedure [11]. A design-to-cost approach may be used to maintain the sustainability of producing 3D-printed simulators for training purposes [12].

The purpose of this technical report is to describe the delivery of a fundamental skills workshop developed to provide hands-on training to rural and remote healthcare professionals, which employs 3D printed simulators purposefully designed following design-to-cost principles. This technical report details the curriculum prepared for a three-hour long workshop that was delivered at a course for rural healthcare professionals in Ottawa, Canada. The workshop provides training on four technical skills using 3D-printed simulators that were curated into working stations for the conference. This report describes the content and delivery of training at each of the four stations (i.e., four technical skills), along with feedback acquired from participants, instructors, and observations, regarding the simulators and overall training process.

## Technical report

The workshop described in this technical report was delivered during the 29^th^ Annual Rural and Remote Medicine Course hosted by the Society of Rural Physicians of Canada (SRPC) in Ottawa, Ontario. The SRPC is a professional organization that represents rural physicians across Canada. Part of SRPC’s mission is to continually promote and deliver rural medical education to rural physicians [13]. The purpose of the Rural and Remote Medicine Course is to provide healthcare professionals and trainees who practice in rural and remote areas with the opportunity to develop and/or maintain clinical competencies, especially those required for high acuity, low occurrence (HALO) events. HALO events are those that do not happen frequently, but when they do occur, can severely impact the patient and require a time-sensitive response [14]. As such, healthcare professionals need to be adequately trained in the skill to perform the procedure promptly if the need arises. The three-hour workshop was developed by a professional team of 10 members from maxSIMhealth Laboratory (www.maxSIMhealth.com), a research laboratory located at Ontario Tech University, Ontario, Canada, in collaboration with representatives of the course’s organizing committee (hereafter referred to as the research team). The focus of the workshop was to provide hands-on training practice using 3D-printed simulators in the context of rural and remote practice. The workshop was attended by 25 SRPC members consisting of medical students, residents, and practicing physicians from across Canada. The specific learning objectives for participants in this workshop were as follows:

1. Demonstrate proficiency when performing the following skills on 3D-printed/silicone models: Suturing, cricothyrotomy, episiotomy, intraosseous infusion (tibial) (IO) 

2. Demonstrate selection of the proper instruments and describe the procedure to perform the skill

3. Demonstrate maintenance of the sterile technique

Workshop process

Participants were self-assigned into four small groups, each around four practice stations: Suturing, cricothyrotomy, episiotomy, and IO. At each station, the participants received instructions from medical expert instructors who are experienced in simulation, as well as they had access to Gamified Educational Networking (GEN).

GEN

**Figure 1:**
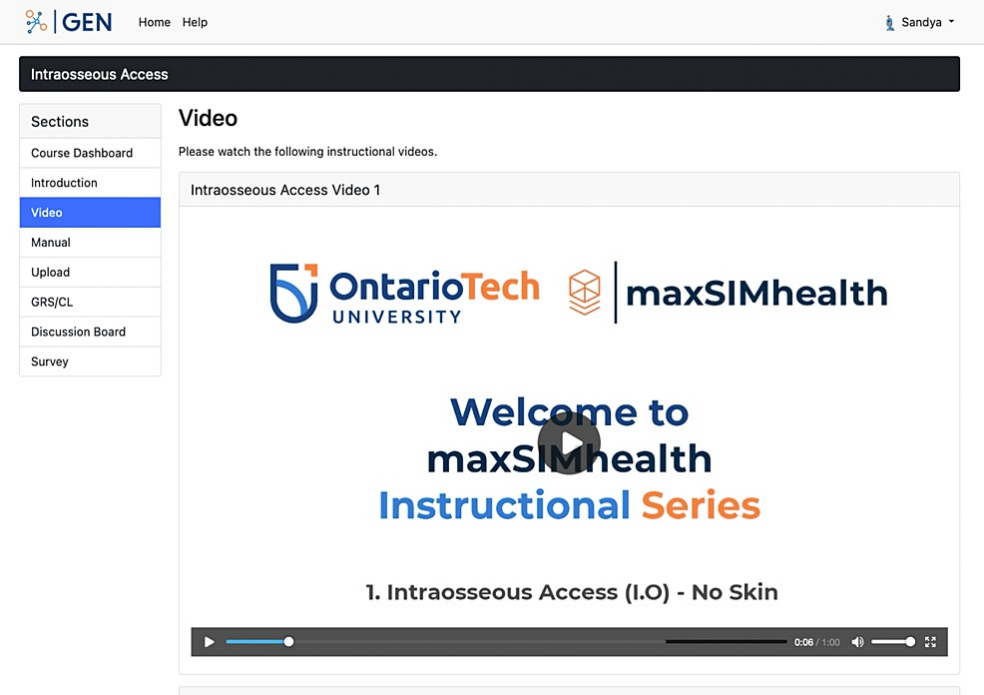
A web page on GEN showing an instructional video for participants to view before practicing on the simulator.

GEN is a learning management system that permits content creation, video uploads, collaborative feedback, and multiple-choice surveys and uses guided learning through discussions [15]. GEN was available at each of the four stations during the workshop for participants. Each of the skills has been allocated a module on GEN where participants were able to watch guided videos of an expert performing the skill (Figure 1) and access a manual presented in PDF format. After watching the videos and reading the manual, participants practiced the skill on a 3D-printed simulator. GEN allows for self-recording, uploading, and sharing videos with peers or a professional for feedback and/or assessment. Additionally, a discussion section is available for participants to ask questions. A guest demo of GEN is available at https://srpc.maxsimgen.com, and the open-source system is available here: https://github.com/maxSIMhealth/GEN. During the workshop all participants were asked to interact with GEN and provide feedback about their experiences. ​​​​​​

Feedback: Overall, GEN was well-received by the attendees of the workshop, who found it to be a useful tool. The participants appreciated the ability to watch the videos prior to practicing on the simulators. A QR code was set up to be scanned for easy access to allow participants to view the content on GEN. After working on the silicone skin suturing pad, one participant suggested uploading the 3D-printed files of the simulator to GEN to allow them to download and print it on their own time with their machine. Another comment received was having the modules accessible to the participants before attending the workshop. We plan to incorporate this as a set of pre- and post-workshop modules. The pre-workshop module would allow the participants to access all the instructional videos and manuals. The participants would be able to watch the instructional videos and read and download the manual, which is consistent with another piece of feedback. After attending the conference, the participants would have access to the post-workshop module that would allow them to upload videos of themselves practicing the skills for evaluation and feedback. Both the pre- and post-workshop modules would have a discussion section for the learners to ask their questions and comment on any concerns. 

The three-hour workshop was divided into four 40-minute sessions. After each session, participants rotated to the next station to practice another one of the four skills. Participants were also provided with the opportunity to explore GEN and more complex simulators such as needle decompression, intravascular access, and the SIMbox. An experienced healthcare professional was designated as an instructor for each skill to deliver instructions and provide feedback to participants. Using a blended learning approach, each station followed a general structure for the training of the skills. Starting with didactic lectures, instructors shared basic knowledge and information with participants to understand how the procedure is performed. Following this was expert demonstrations where the instructor performed the skill on the simulator to help participants identify the step-by-step process to perform the skill. After watching the instructor demonstrate the procedure, an active learning approach was employed where participants were able to perform the procedure on an identical simulator. Participants were given the opportunity to repeatedly practice the procedure until they felt comfortable performing the skill to develop a high degree of proficiency by applying the knowledge and feedback provided by the instructor [16]. 

Peer-assisted learning (PAL) strategies were applied at each station to support the skill development of participants. PAL is the use of active learning with the support of peers to assist in teaching and improving retention by having students teach one another to deepen their learning and understanding of a topic [17]. The simplified instructor-to-student ratio for training on technical skills is predicted to be one instructor for every four students (1:4) taught to achieve an optimal amount of learning [18]. Using this approach to teaching, the research team adapted the ratio to have one instructor present for every eight participants (1:8) and incorporated PAL to offset the variance in the ratio. Therefore, at each station, there was one instructor who provided information, demonstrations, and individual feedback, and eight learners that would pair up to use PAL when practicing the skill.

Station 1: Suturing

**Figure 2:**
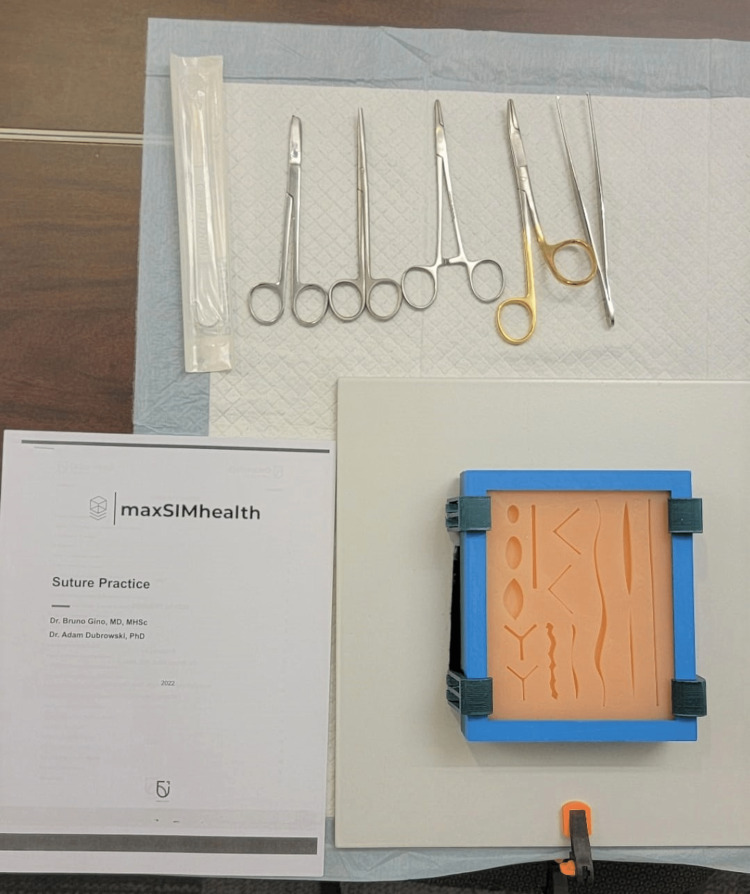
Equipment setup of the suturing station for an individual participant.

**Figure 3:**
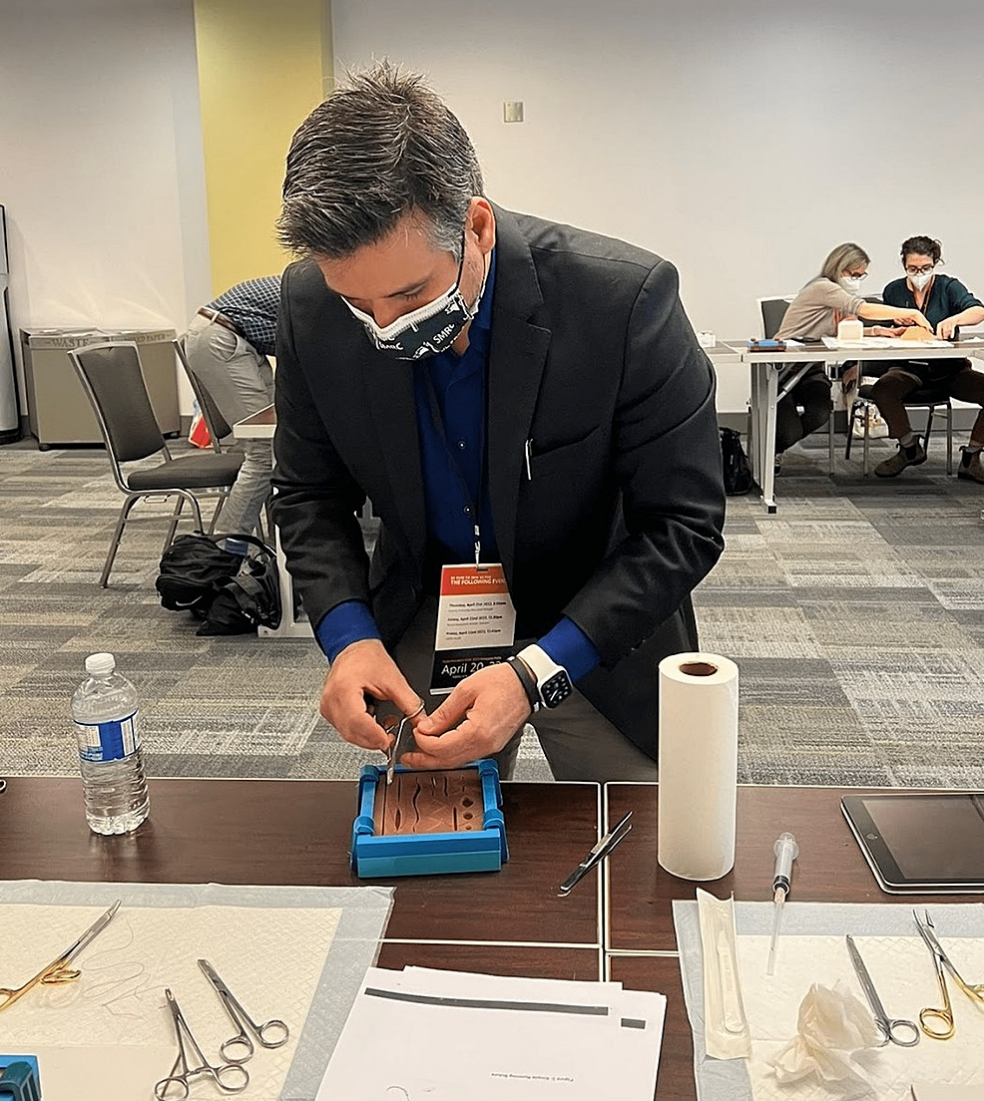
Instructor demonstrating one of the suturing skills for participants.

Content and delivery: A silicone skin suturing pad was used to train participants on suturing during the workshop [19]. The simulators came in different skin tones and consisted of 14 different cuts of varying shapes, sizes, and depths. The instruments to perform the suturing skill were supplied at the station with the simulator and a printout of a manual describing seven types of sutures (Figure 2). In addition, an instructional video for each type of suture was hosted on GEN demonstrating the surgical technique and was made accessible to participants at the station as well. Clinical experts trained in the suturing skill used the manual to guide participants at the station and demonstrated seven sutures: simple interrupted suture; simple running suture; simple running locking suture; vertical mattress suture; horizontal mattress suture (interrupted); horizontal mattress suture (continuous); and subcuticular suture (Figure 3). Following the expert demonstration, participants were given an opportunity to attempt the skill as many times as they desired. The 3D-printed files for the suturing pad mold and instructional videos for each of the seven suture techniques presented at this workshop have been made publicly available on GitHub and can be accessed using the following link: https://github.com/maxSIMhealth/maxSIM_Suturing_v1.0.

Feedback: Verbal feedback from participants and instructors for the suturing station was gathered in the form of written field notes. In general, the feedback was positive and constructive. Most participants sought out the instructor during the session to ask more about each of the seven sutures using the simulators and asked questions about the manual and video. Some even requested access to the manual and video for future reference. Based on this, instructors felt that the participants were engaged in the station and were interested in the simulators and learning the suturing technique. The main criticism of the station came from the instructor - they believed that more hands-on time, as opposed to didactic teaching, should be allocated to the station to explore the content in greater detail as all the content cannot be covered in a three-hour-long workshop. The instructor provided minor feedback on the simulator explaining that the thread would escape the silicone at times because it was not strong enough, and it was slightly more difficult to do a deeper stitch on the silicone.

Station 2: Cricothyroidotomy

**Figure 4:**
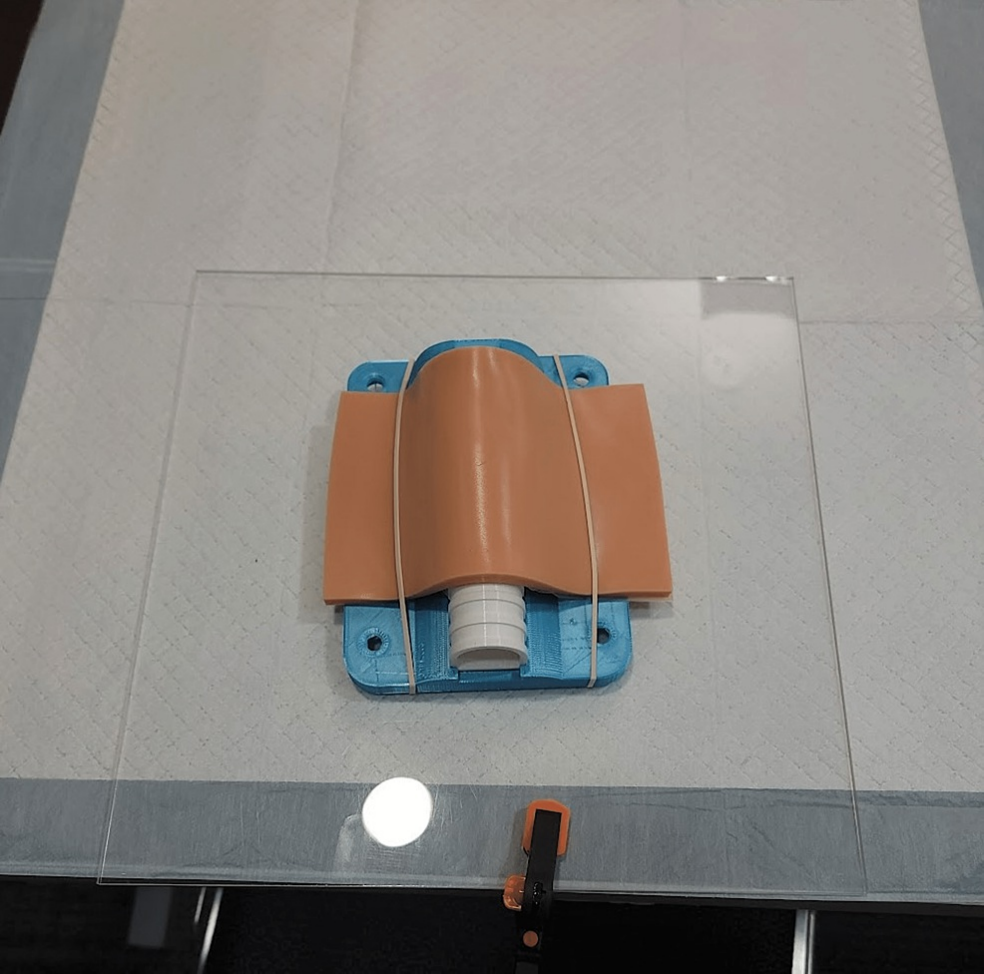
The cricothyroidotomy simulator that participants used to practice the skill.

**Figure 5:**
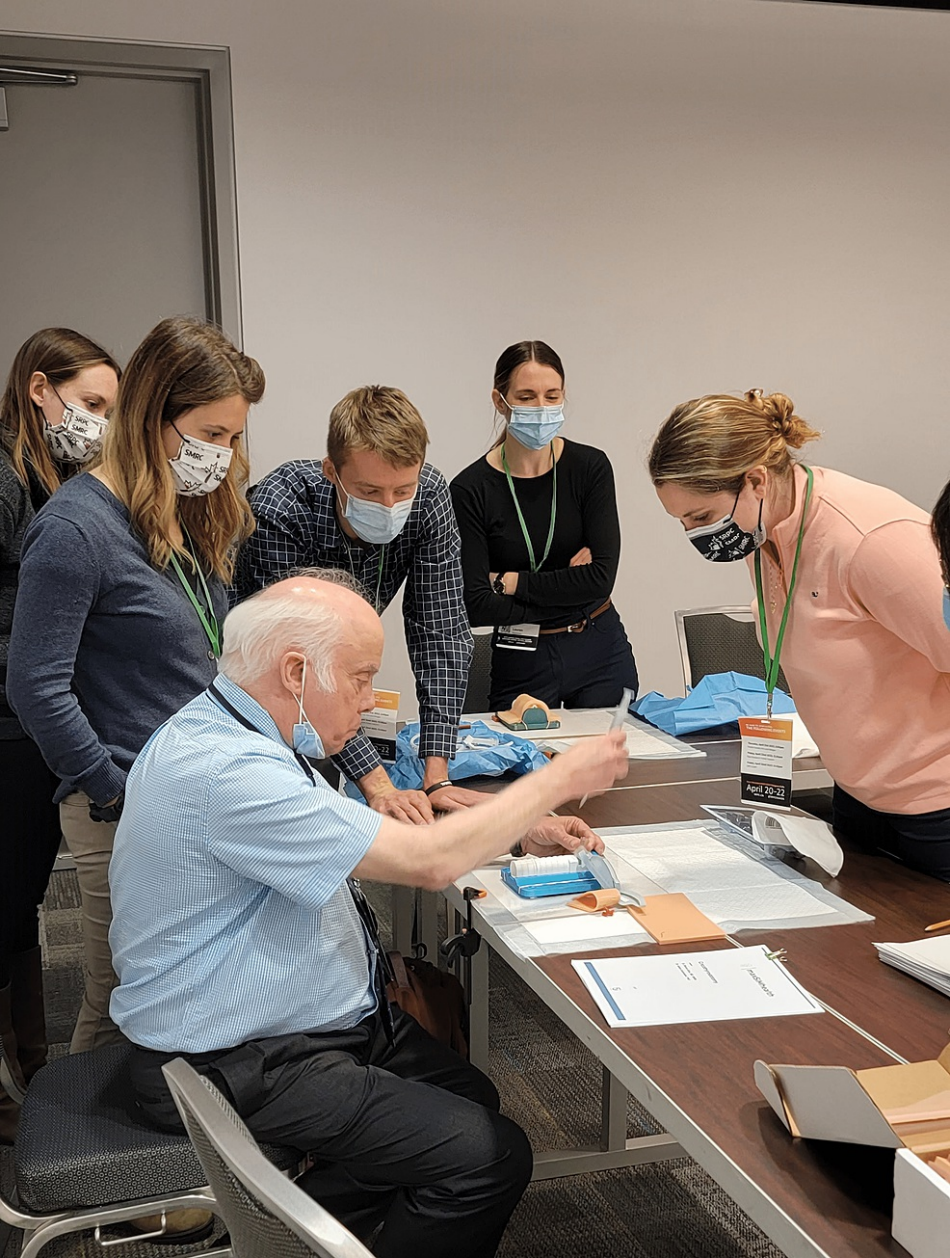
Instructor demonstrating the cricothyroidotomy skill for participants using the simulator.

Content and delivery: The cricothyroidotomy station was organized to teach participants how to perform a cricothyroidotomy procedure, specifically the surgical technique and the needle puncture technique, including the Seldinger technique, on a cricothyroidotomy simulator [20]. The equipment was set up at the station with the cricothyroidotomy simulator for the participants (Figure 4). An instructional video hosted on GEN and a printout of a manual describing the two cricothyroidotomy techniques were also made available at the station. Clinical experts demonstrated both cricothyroidotomy techniques to the participants using the equipment provided (Figure 5). Afterward, participants were given an opportunity to attempt the skill as many times as they desired using the same equipment with guidance from the clinical experts, instructional videos on GEN, and a document outlining all of the steps. The 3D-printed files for the cricothyroidotomy simulator and instructional videos for the two cricothyroidotomy techniques taught during this workshop have been made publicly available on GitHub and can be accessed using the following link: https://github.com/maxSIMhealth/maxSIM_Crico_v1.0.

Feedback: Following the sessions, a few participants and instructors engaged with the research team to offer their experiences relating to the cricothyroidotomy station. The main comment received from participants was to add blood flow throughout the simulator so that when cut, simulated blood can flow out just as it would when performed on an actual patient.

Overall, the instructor believed that the cricothyroidotomy simulator was satisfactory in terms of a training tool, and noted that the participants were excited to try the simulator. While a presentation was prepared with the intent of needing to demonstrate the skill four times (once with each group), the instructor found that the presentation was not needed as participants were more interested in hands-on practice on the simulator. Because of the complexity of the skill, the instructors noticed that an additional instructor/assistant at the station would help with the demonstration. With respect to the simulator itself, the instructor liked the simplicity of it but suggested that the skin layer be thinner. The thickness of the skin layer for the cricothyroidotomy simulator, as with other simulators developed in the maxSIMhealth lab, was determined through an iterative process of consultations between the development and clinical team and participant feedback [20-21].

Station 3: Episiotomy

**Figure 6:**
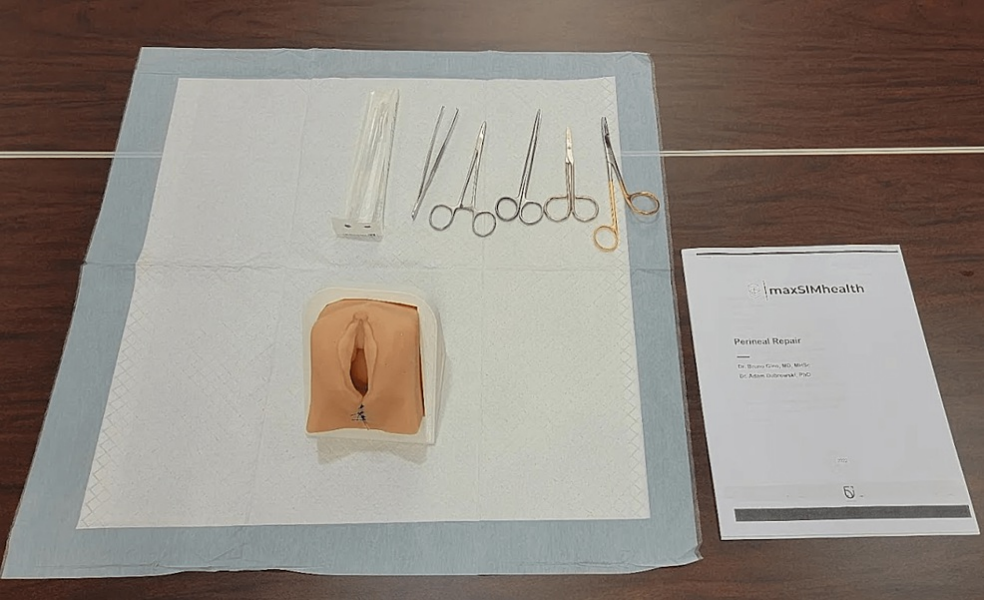
Equipment setup of the episiotomy station for an individual participant.

**Figure 7:**
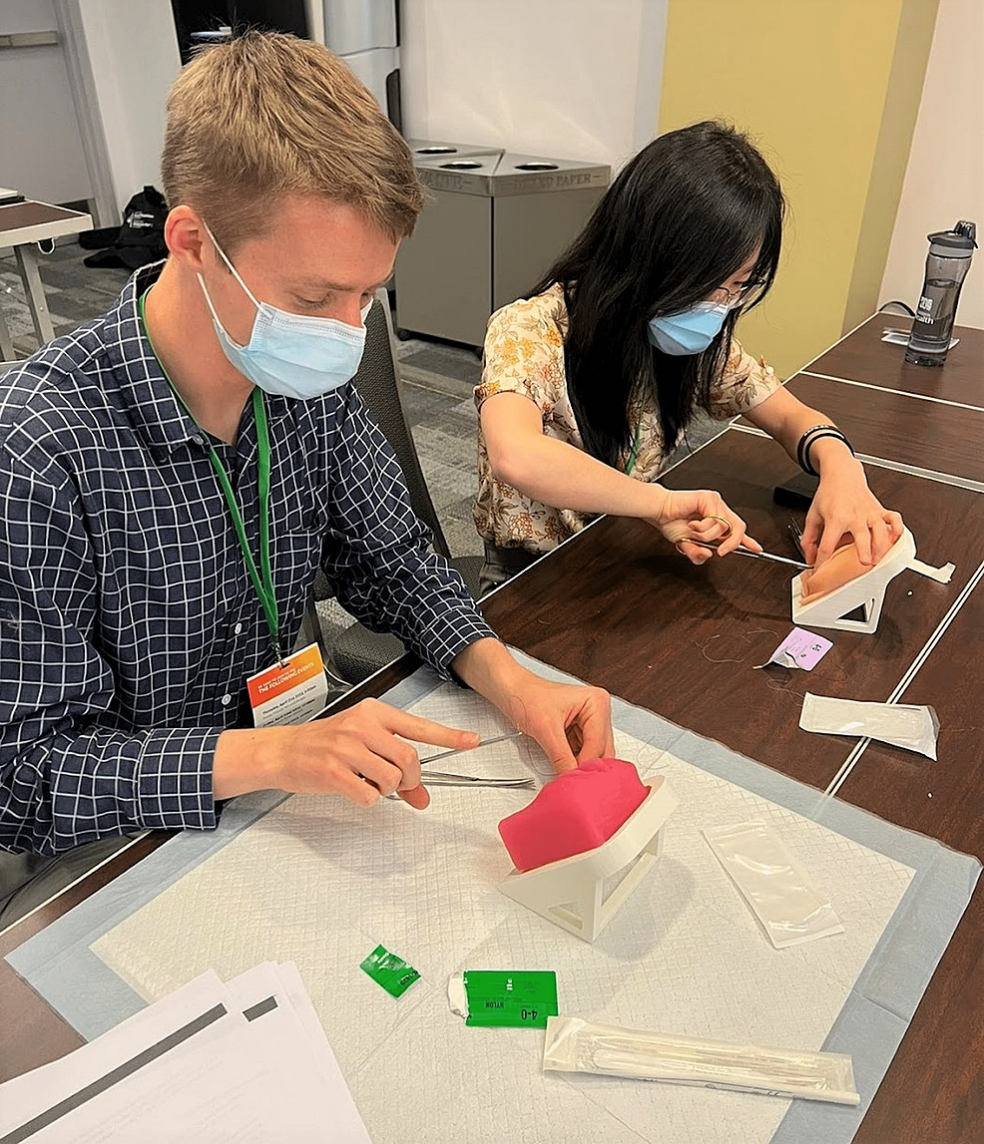
Participants practicing the episiotomy technique on the simulator.

Content and delivery: The episiotomy station was organized to teach participants how to perform an episiotomy of various degrees of tears (first to a fourth degree) on an episiotomy simulator [22]. The equipment at the station included the episiotomy simulator, instruments to perform the technique, and an instruction manual describing the episiotomy technique (Figure 6). An instructional video hosted on GEN was also made available at the station. Clinical experts demonstrated the episiotomy technique to various degrees to the participants using the equipment provided. Afterward, participants were given an opportunity to attempt the skill as many times as they desired using the same equipment, with guidance from the clinical experts, instructional videos on GEN, and a document outlining all the steps (Figure 7). The 3D-printed files for the episiotomy simulator and instructional video showing the episiotomy technique used for this workshop have been made publicly available on GitHub and can be accessed using the following link: https://github.com/maxSIMhealth/maxSIM_episiotomy_v1.0.

Feedback: The instructor for the episiotomy station found that the episiotomy simulator being held on a 3D-printed stand was natural to the positioning and worked well for practice. The instructors did not have access to the models before the workshop itself and felt that coming into the station, there was not enough preparation done to brief them on the organization and schedule of the station before taking part. The instructor suggested these components be incorporated into future workshops for better preparedness. During the station, the instructor felt that how the procedure was being taught made it difficult to manage the learning process of all participants at once since some participants were at different steps in the process, delaying the training. Ultimately, the instructor felt that the station could have benefitted from more time and structure. For example, the instructor suggested that it would be beneficial to the learners to have time to go through the educational content before the session (i.e., instructions, techniques, materials, demonstrations, and videos), then allow for ample time to practice. 

The instructor identified that not all the instruments provided were correct for this procedure, and there were not enough supplies. They advised obtaining a sufficient supply of scissors, forceps, and larger and more curved needles for the future to achieve greater realism in the scenario being simulated through the station. 

With regards to the physical perineal simulator itself, the instructor suggested making the silicone softer and having the opening of the vagina larger. Additionally, it was noted that adding muscle layers to the model would create a more anatomically accurate simulation. Despite these recommendations, the instructor rated the importance of this particular hands-on station as high, emphasizing that in rural and remote settings, not many opportunities for practice exist currently. Recommendations for future workshops suggested that training could be conducted with the simulator as practice first, followed by the opportunity for participants to test their learning on real tissues. This approach to learning could reinforce the training for the learner and increase transferability in a real clinical setting.

Station 4: Intraosseous infusion

**Figure 8:**
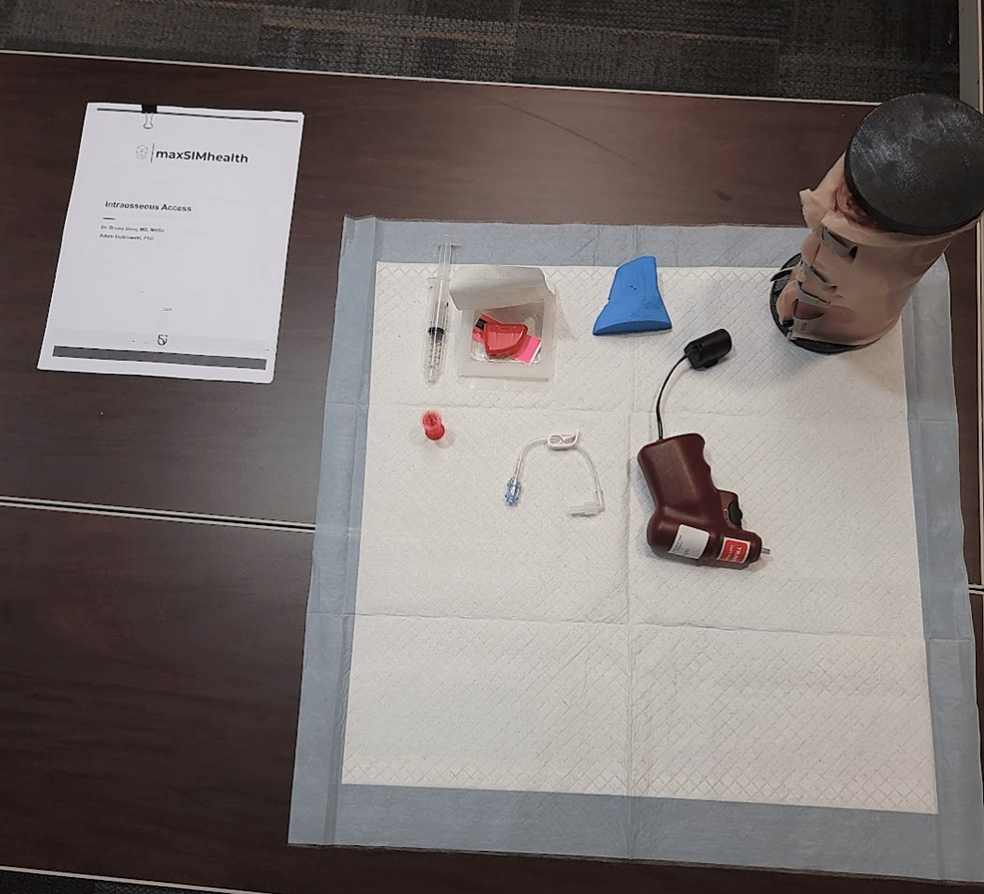
Equipment setup of the intraosseous infusion station for an individual participant.

**Figure 9:**
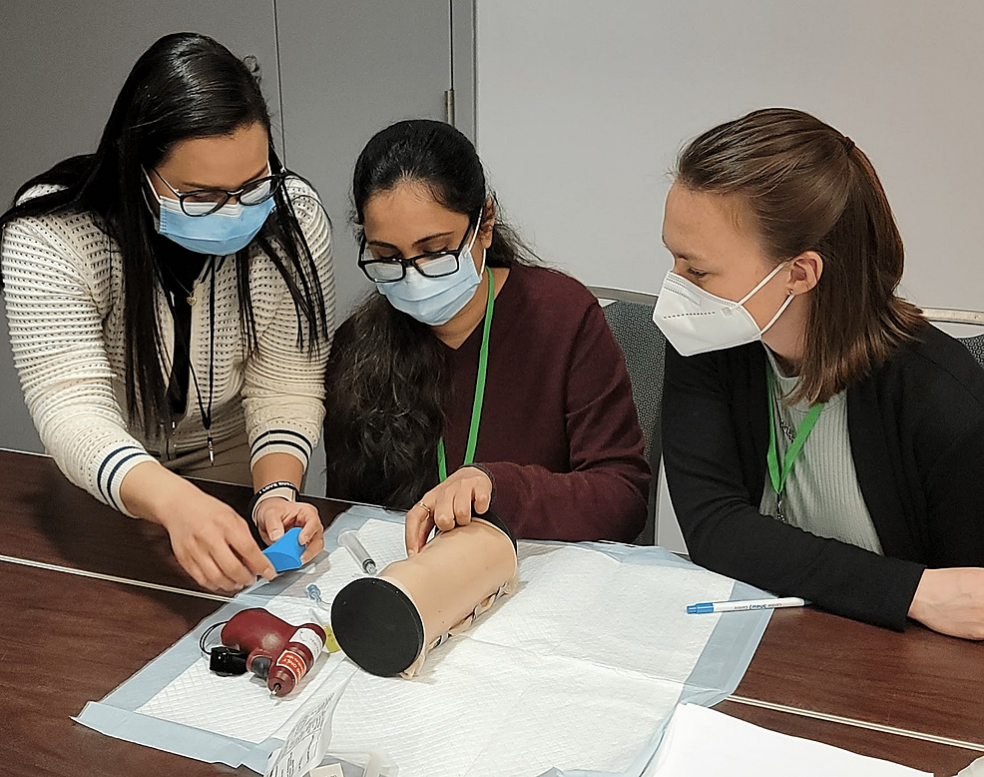
Instructor training participants on the IO technique using the simulator.

Content and delivery: A 3D-printed adult proximal tibia IO simulator, maxSIMIO, was utilized to teach rural and remote doctors and trainees the IO technique at one of the workshop stations [23]. The equipment was arranged at the station for participants with the simulator and a printout of an instruction manual describing how to perform the IO skill (Figure 8). An instructional video hosted on GEN was also made available at the station. Clinical experts demonstrated the IO technique to the participants using the equipment at the station and highlighted appropriate landmarking, needle insertion, and the attachment of a catheter hub (Figure 9). Afterward, participants were given an opportunity to attempt the skill as many times as they desired using the same equipment. The 3D-printed files for the IO simulator and instructional videos showcasing the IO technique used for this workshop have been made publicly available on GitHub and can be accessed using the following link: https://github.com/maxSIMhealth/maxSIMIOv1.0.

Feedback: The participants expressed that the simulator was helpful in learning and teaching the IO technique. Instructors also pointed out that the video that was played for the participants was very helpful. Additionally, instructors described how the simulator could be improved, regarding stability and realism. To physically stabilize the simulator while performing the IO technique, it was recommended that the simulator be made flat on one side. In terms of its realism, the instructors suggested the following: 1) the skin is made and secured to the simulator better to show the contours of the muscle and bones of an adult leg, 2) the bones within the simulator are made fixed to prevent movement during the procedure, 3) the skin is made to look more anatomically accurate (i.e., texture, color, and features such as pores and hair), and 4) the patella is made slightly larger and denser in order to improve the feeling of resistance during the procedure (i.e., “pop” achieved during the procedure is more apparent). Lastly, one instructor suggested that the simulator should show blood coming through the syringe once the bone marrow is met in the procedure if it is feasible from a manufacturing standpoint. In summary, the instructors pinpointed key areas in which the simulations could be fine-tuned to improve the overall experience in performing the IO procedure.

Concerning the delivery of the station, instructors thought it was well formulated and coordinated. In terms of pedagogy, instructors felt that there was a good number of learners at the station at any given point in time to train, and the time provided for training was sufficient. Generally, participants at the station showed more interest in the simulators and relied heavily on the simulators to pose questions to the instructors about the procedure. Regarding areas for improvement, instructors advised including extra materials like stabilizers, needles, extensions, and syringes to increase authenticity in the procedure. It was noticed that at the station, some participants had to wait to have a turn using the simulator and an IO drill as they were limited in numbers compared. One possible innovation to offset the shortage of drills is to use a small, commercially available power screwdriver or drill and use a 3D-printed drill adaptor. The research team has developed and tested one, which is available here: https://github.com/maxSIMhealth/EZIOdrill. Consequently, some participants missed instructions during this waiting period. Lastly, although the video that the instructor showed of the procedure was very helpful according to the instructor, the sounds coming through the device provided to show the video was quiet and thus, not very well received by the participants. Overall, both feedback received for the IO station was valuable in developing our simulator as an effective tool to learn the IO technique.

Display station

**Figure 10:**
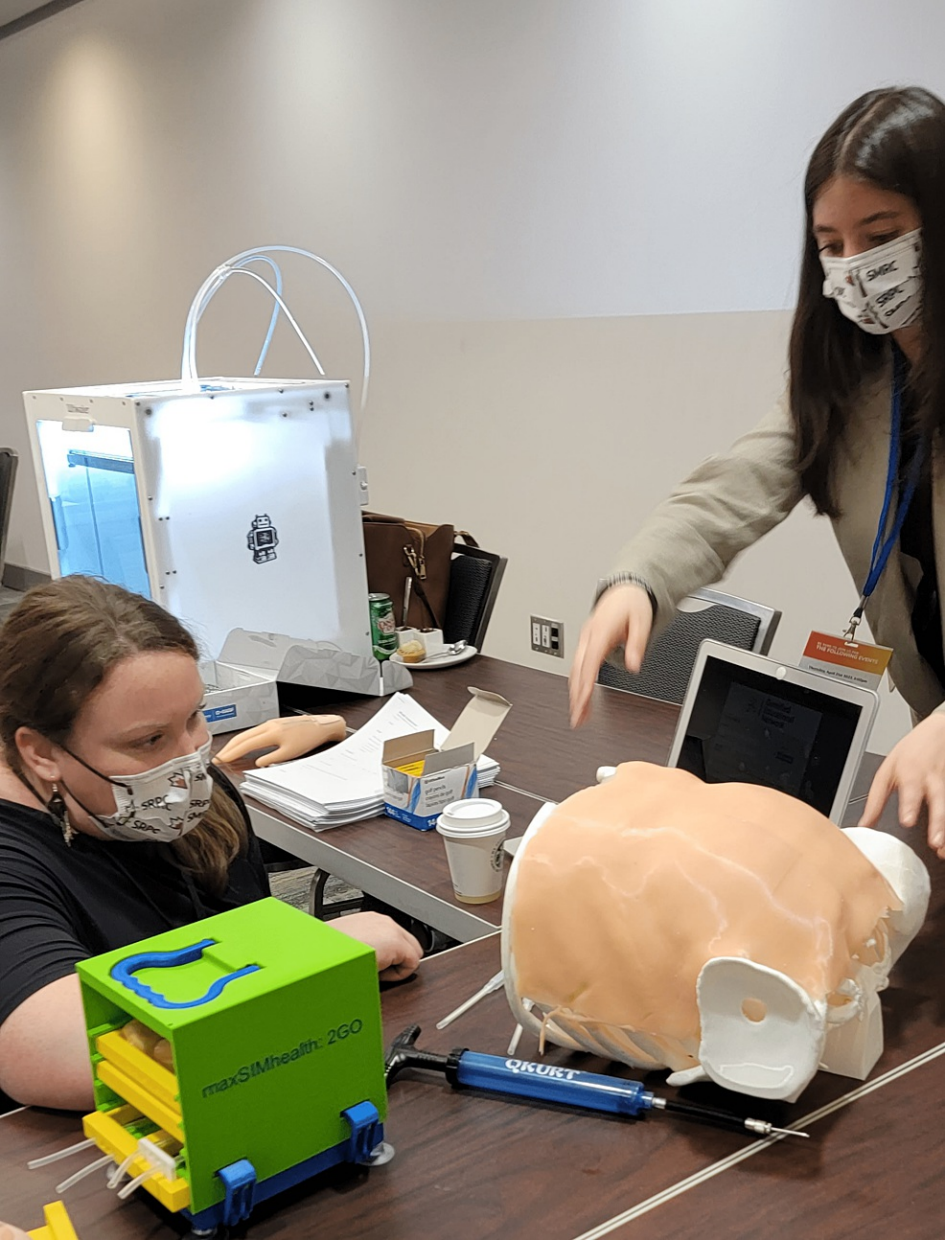
A participant viewing a simulator at the display station guided by one of the research team members.

Content and delivery: Along with the four skill stations, a fifth station, the display station, was presented at the workshop. This station featured additional simulators, a 3D printer, and a computer with access to GEN for participants to view in between rotations and as they walked around the room (Figure 10). The display station included a hand model to practice intravascular access, a needle decompression model, and the SIMbox, a travel-friendly 3D-printed box that contains five training skill simulators: intramuscular injection, IO, knot tying, suturing, and wound cleaning. In addition to the simulators, a 3D printer was placed at the display station and was set up to print a maxSIMhealth keychain design during the workshop to allow participants to view the method by which the simulators are printed. The purpose of this station was to display the various ways that 3D printing could be used to develop simulators for medical training purposes and to receive feedback from participants on the functional use and anatomical accuracy of the models to improve future versions of the model.

Feedback: Feedback for simulators at the display station was acquired through field notes collected by the research team. Overall observations regarding the display station highlighted that participants were not as interested in testing out the IV or needle decompression models. The majority of the participants simply observed and/or touched the simulators. Participants showed more interest in viewing the 3D printer and the process of how the simulators are made. Regarding the IV simulator, one participant mentioned that it was one of the most realistic feeling IV models they had used. A suggested improvement was to incorporate blood flow through it with a catheter to insert into it instead of just a needle. One participant mentioned that the needle decompression model provided is better than another model that they have used before. One suggestion provided for this model is to insert something between the ribs to mimic intercostal muscles. Points of feedback acquired for the SIMbox suggested the need to add a door to the front of the box and make the components of the simulator the appropriate size to fit within the box. Additionally, to acquire more detailed feedback, one suggestion was to have the instructor/evaluator spend more time with the box to test out each component.

## Discussion

This technical report describes the structure, process, and delivery of a fundamental skills workshop. This workshop was delivered using 3D-printed simulators at a course for rural and remote healthcare professionals. The objectives of this workshop were to enable the participants to become familiar with techniques and instruments used in rural and remote practice. Taking four clinical skills: suturing, cricothyrotomy, episiotomy, and intraosseous infusion, attendees had the opportunity to learn, apply, and repeat the procedure at different stations of the workshop to advance their proficiency in performing the skill.

After the conclusion of the course, an online survey was circulated by the course’s planning committee for each workshop to collect feedback from the workshop participants. Participants of the workshop strongly agreed that the training instructors had the appropriate knowledge of the subject matter and that the information was presented clearly. Participants agreed or strongly agreed that the information presented was useful and met the stated learning objectives. One participant elaborated in the comments highlighting that they found the three-hour long workshop to be too long and suggested breaking it down into three smaller sessions. 

In addition to the online survey, verbal feedback was collected from the participants and instructors at each station in the format of field notes. Overall, the instructors felt that the workshop went relatively well and was well organized and dynamic. The feedback highlighted that 3D printing can allow learners to print the model and practice on their own time in their own emergency room if they have access to a 3D printer. Feedback on the structure of learning at each station highlighted that showing videos, having the instructor demonstrate, and then having the learner practice while the instructor provides feedback is optimal for the learner to reproduce what they have learned. In addition, skills training such as this needs to occur regularly, as a three-hour workshop may not be sufficient for participants to become proficient in the skill [3]. Skills such as episiotomy and cricothyroidotomy are categorized under HALO events where healthcare professionals will rarely need to perform the procedure, but due to the time-sensitivity of the event, they may be the only person who can perform the procedure without the ability to ask questions [14]. Therefore, it is important to have a lot of practice as HALO events cannot be predicted. Simulation is one way of providing hands-on experiences to the learners for HALO skills; however, the costs of simulators and access to instructions and feedback are two barriers that rural and remote practitioners face [3,7]. The feedback from the workshop clearly supports the idea of using alternative means to manufacture simulators in a just-in-time, locally, at a reduced cost fashion using 3D printing technologies. 

As it relates to the physical simulators, there may be a need to switch from a design-to-cost approach to a design-to-value approach. The design-to-value approach may be guided by learning objectives that the simulator is designed to deliver and therefore can be referred to as a design-to-learning objective approach. A few of the suggestions provided may not substantially increase the cost of producing the simulator. However, the research and development process to incorporate the feedback in making specific simulators more realistic when performing the procedure may require a design-to-value approach. 

Furthermore, the feedback was very positive about using learning management systems, such as GEN, to provide instructions and feedback to remote learners. In addition, as one participant suggested, the use of such a learning management system as a pre-learning activity can help to reduce the didactic component of the workshop and allow for more hands-on training. That is, by incorporating observations and instructor and participant feedback, the didactic components of the workshop will be minimized, and GEN would be made available to all the participants two weeks before the workshop to sign up on the platform and view the instructional materials and videos. After doing so, the participants would receive a certificate for reviewing the instructional material.

## Conclusions

The fundamental skills workshop is a necessary training opportunity for healthcare professionals practicing in rural and remote communities to learn and review their training on important technical skills both synchronously and asynchronously. The use of 3D-printed simulators to provide hands-on practice is a feasible and sustainable method to develop skill-based competencies. This, in conjunction with the use of a learning management system, could provide a repository to learn new materials, record and upload personal practice sessions, review other learners' practice sessions, and collaborate and provide feedback to other learners. The feedback collected from participants, instructors, and observations on the delivery of this workshop will help improve the structure and training provided to learners. The delivery of this workshop hosted annually at a course is an ideal solution to ensure parsimonious delivery of simulation training for rural and remote healthcare professionals.

## References

[1] (2022). The College of Family Physicians of Canada: Review of family medicine within rural and remote Canada: Education, practice, and policy. https://www.cfpc.ca/CFPC/media/Resources/Rural-Practice/ARFM_BackgroundPaper_Eng_WEB_FINAL.pdf.

[2] Williams KL, Renouf TS, Dubrowski A (2020). Pitfalls in emergency medicine: survey-based identification of learning objectives for targeted simulation curricula by emergency department staff. Cureus.

[3] Ahmed K, Wang TT, Ashrafian H, Layer GT, Darzi A, Athanasiou T (2013). The effectiveness of continuing medical education for specialist recertification. Can Urol Assoc J.

[4] So HY, Chen PP, Wong GK, Chan TT (2019). Simulation in medical education. J R Coll Physicians Edinb.

[5] Aimar A, Palermo A, Innocenti B (2019). The role of 3D printing in medical applications: A state of the art. J Healthc Eng.

[6] Patey C, Norman P, Bishop N, Bartellas M, Dubrowski A (2018). Development, evaluation, and implementation of a new 3D printed tongue depressor dispenser. Cureus.

[7] Kholgh Eshkalak S, Rezvani Ghomi E, Dai Y, Choudhury D, Ramakrishna S (2020). The role of three-dimensional printing in healthcare and medicine. Mater Des.

[8] Kothari LG, Shah K, Barach P (2017). Simulation based medical education in graduate medical education training and assessment programs. Prog Pediatr Cardiol.

[9] Leung G, Pickett AT, Bartellas M, Milin A, Bromwich M, Shorr R, Caulley L (2022). Systematic review and meta-analysis of 3D-printing in otolaryngology education. Int J Pediatr Otorhinolaryngol.

[10] DiMaggio PJ, Waer AL, Desmarais TJ (2010). The use of a lightly preserved cadaver and full thickness pig skin to teach technical skills on the surgery clerkship--a response to the economic pressures facing academic medicine today. Am J Surg.

[11] (2022). Spartan: Design for cost from the perspective of competitive advantage. http://spartan.ac.brocku.ca/~pscarbrough/dfca1stmods/dfc/dfcst.html.

[12] Barth B, Arutiunian A, Micallef J (2022). From centralized to decentralized model of simulation-based education: curricular integration of take-home simulators in nursing education. Cureus.

[13] (2022). Society of Rural Physicians of Canada: About the society. https://srpc.ca/about-the-srpc.

[14] Chiniara G, Cole G, Brisbin K, Huffman D, Cragg B, Lamacchia M, Norman D (2013). Simulation in healthcare: a taxonomy and a conceptual framework for instructional design and media selection. Med Teach.

[15] Torres A, Kapralos B, Uribe-Quevedo A, Quero EZ, Dubrowski Dubrowski (2020266275). A: A gamified educational network for collaborative learning. Internet of Things, Infrastructures and Mobile Applications.

[16] Sigalet E, Wishart I, Lufesi N, Haji F, Dubrowski A (2017). The “empty chairs” approach to learning: simulation-based train the trainer program in Mzuzu, Malawi. Cureus.

[17] Jauregui J, Bright S, Strote J, Shandro J (2018). A novel approach to medical student peer-assisted learning through case-based simulations. West J Emerg Med.

[18] Dubrowski A, MacRae H (2006). Randomised, controlled study investigating the optimal instructor: student ratios for teaching suturing skills. Med Educ.

[19] Gallagher PO, Bishop N, Dubrowski A (2020). Investigating the perceived efficacy of a silicone suturing task trainer using input from novice medical trainees. Cureus.

[20] Doucet G, Ryan S, Bartellas M, Parsons M, Dubrowski A, Renouf T (2017). Modelling and manufacturing of a 3D printed trachea for cricothyroidotomy simulation. Cureus.

[21] Habti M, Bénard F, Arutiunian A (2021). Development and learner-based assessment of a novel, customized, 3D printed small bowel simulator for hand-sewn anastomosis training. Cureus.

[22] Goudie C, Shanahan J, Gill A, Murphy D, Dubrowski A (2018). Investigating the efficacy of anatomical silicone models developed from a 3D printed mold for perineal repair suturing simulation. Cureus.

[23] Sivanathan M, Micallef J, Clarke KM (2022). The development and initial end-point user feedback of a 3D-printed adult proximal tibia IO simulator. Cureus.

